# Potential Effect of Trigonella Microgreens on Functional Parameters of HUVEC Cells *in vitro*

**DOI:** 10.33549/physiolres.935643

**Published:** 2025-12-01

**Authors:** Tomas JAMBOR, Zofia GOC, Katarina TOKAROVA, Norbert LUKAC

**Affiliations:** 1 Institute of Applied Biology, Faculty of Biotechnology and Food Sciences, Slovak University of Agriculture in Nitra, Nitra, Slovakia; 2 Institute of Biology and Earth Sciences, Faculty of Exact and Natural Science, University of the National Education Commission, Krakow, Poland

**Keywords:** *Trigonella-foenum graecum L.*, Microgreens, Cytokines, HUVEC cells

## Abstract

The growing popularity of microgreens is due to several health-promoting effects. Current evidence suggests a reduction in the risk of cardiovascular and neurodegenerative diseases, or anti-carcinogenic and anti-inflammatory effects. However, *in vitro* studies investigating cellular changes and molecular mechanisms are limited. Therefore, the use of various cell lines is required for a better understanding of microgreens’ effects. In this study, the effect of *Trigonella-foenum graecum L*. microgreens (10–300 μg/mL) extract on morphological and functional changes in HUVEC cells were investigated. Basic cellular parameters such as mitochondrial activity (MTT assay), cell membrane integrity (CFDA-AM assay), and lysosomal activity (NR uptake) were evaluated after 24 h exposure to experimental ethanolic extract. In addition, the release of cytokine IL-6 was measured. Results revealed significant changes *(p<0.05; p<0.01)* in mitochondrial activity, followed by no defect in cell membrane integrity and non-significant changes in lysosomal activity of HUVEC cells. At the same time, some experimental doses of *Trigonella* slightly modulate IL-6 release, but showed no significant changes after 24 h exposure. Overall, our pilot study suggests the potential of Trigonella microgreens to modulate mitochondrial activity of HUVEC cells, without significant changes in cell membrane integrity, lysosomal activity, or IL-6 release.

Chronic diseases are one of the major health problems in Europe, with the incidence of heart attacks, cancer, asthma and obstructive pulmonary disease constantly increasing. Immunodeficiency and inflammation play an important role in the pathogenesis of these chronic as well as acute diseases [1]. A recent research study has identified cellular and molecular mechanisms that operate on the NF-kB inflammatory pathway via A20/TNFAIP3, and they are involved in autoimmune diseases [2]. In addition, various inflammatory cytokines, such as TNF-α, IFN-γ, CCL5, IL-12 or IL-6, are clearly involved in the pathogenesis of chronic diseases [3,4]. Considering the above facts, it seems that the proposed experimental model of HUVEC cells can be used as a tool for monitoring possible pathological and inflammatory changes, similar to a recent scientific study [5].

Accumulated data also suggest that regular consumption of plant resources, rich in phytonutrients, may reduce the risk of chronic diseases incidence, and could have significant immunomodulatory potential. [6]. From this perspective, microgreens grown under strictly regulated conditions without external stress factors can be considered an excellent source of phytonutrients. The presence of vitamins, anthocyanins, glucosinolates, macroelements and other health-promoting micron-trients makes microgreens a significantly more potent source of beneficial effects on human health than their mature counterparts [7,6]. Recent studies report that growing *Trigonella foenum-graecum L*. as a microgreen stimulates the accumulation of health-promoting phytonutrients, increases the content of various phenolic acids (gallic acid, p-coumaric acid), amino acids (isoleucine, phenylalanine) and other alkaloids and terpenoids as well. There is an assumption that different biologically active molecules in Trigonella could affect morphological and functional parameters of HUVEC cells, which are directly related to changes in secretory activity and cytokine release [6,8]. Based on a previous study [7] the authors designed a panel of experimental concentrations that can induce changes in cell proliferation at the intracellular level and, together with other studies, indicate changes in mitochondrial activity and secretory activity. Microgreens have not yet revealed their unique potential and experimental studies investigating direct effects on cellular systems are currently lacking. The results of this pilot study could indicate the potential of *Trigonella* to affect the functional activity of HUVEC cells, which are suitable alternative models for the research of chronic diseases and monitoring the physiology of inflammation in future studies. The aim of our in vitro study was therefore to investigate the potential of the microgreen *Trigonella foenum-graecum L*. and its impact on mitochondrial activity, cell membrane integrity, and lysosomal activity of human umbilical vein endothelial cells (HUVEC). In addition, the secretory activity and release of IL-6 were also analyzed.

The human umbilical vein endothelial cells (HUVEC) were purchased from American Type Culture Collection (ATCC, CRL-1730, Manassas, VA, USA), and cultured in endothelial cells grown medium (Medium199; Sigma-Aldrich, St. Louis, MO, USA) supplemented with 20 % of foetal bovine serum (FBS; BiochromAG, Berlin, Germany); 3 % of endothelial cell growth factor (ECGS; Corning+, NY USA), 1 % of L-glutamine (Sigma-Aldrich, St. Louis, MO, USA), 1 % of penicillin/streptomycin solution (Sigma-Aldrich, St. Louis, MO, USA), and 1 % of heparin (Thermo Fisher Scientific Inc, Waltham, MA, USA) as well. Cells were cultivated in strictly controlled conditions in CO_2_ incubator with 95 % of humanified atmosphere, at 37 °C, with 5 % CO_2_. Cells were regularly screened for microbial contamination by the PlasmoTestTM (InvivoGen Inc, San Diego, CA, USA). Cells were seeded in 96-well plates at 1 × 10^4^ cells/well and pre-cultured for 24 h. Afterwards, culture media was changed to include experimental concentrations of *Trigonella foenum-graecum L*. microgreen extract (from 10 to 300 μg/mL) and remained cultured during the next 24 h. The panel of experimental doses was set up according to previous study with different cellular model, including positive control group - cells treated by zymosan. For extract preparation, one gram of fresh Trigonella microgreens was extracted by adding 10 mL of 80 % (v/v) aqueous ethanol (EtOH; Sigma, St. Louis, MO, USA) for 12 h with constant horizontal shaking at room temperature. Subsequently, the crude extract was centrifuged (9,000 rpm, 4 °C, and 5 min), the supernatant was collected, filtered through Q-Max RR syringe filter (0.22 μm PVDF, diameter: 25 mm; Frisenette ApS, Denmark), and stored in the dark at 4 °C until used in the experiment. Crude extract was dissolved in dimethyl sulfoxide (DMSO, Sigma, St. Louis, MO, USA), and the concentration did not exceed 0.6 % (v/v) [7,10]. The mitochondrial activity of HUVEC cells was determined by (3–4,5-dimethylthiazol-2-yl)-2,5-diphenyltetrazolium bromide (MTT, Sigma-Aldrich, St. Louis, MO, USA) assay, which relies on the cells ability to convert yellow tetrazolium salt into blue formazan deposits. They were subsequently dissolved by DMSO and read by combined spectro-fluoro luminometer (GloMax® - Multi+ Microplate Multimode reader with Instinct®; Promega Corporation, Madison, USA) at 570 nm against 620 nm wavelengths. Besides that, the membrane integrity and lysosomal activity of exposed HUVEC cells was evaluated by 5-carboxyfluorescein diacetate, acetoxymethyl ester (CFDA-AM; Thermo Fisher Scientific, Inc Watham, MA, USA) or Neutral Red uptake (NR, Sigma-Aldrich, St. Louis, MO, USA) and measured by spectro-fluoro luminometer GloMax® - Multi+. Human interleukin-6 released to the cell culture media was determined by enzyme-linked immunosorbent assay and ELISA kit (IL-6; MyBioSource, San Diego, USA). The absorbances were read by microplate reader Multiscan FC (Thermo Fisher Scientific Inc, Waltham, MA, USA) with set up wavelength 450 nm. Each experiment was conducted at least three times, using cells from different passages from no. 7 to passage no. 25. Statistical analyses were carried out by using the GraphPad Prism program (version 6.07, GraphPad Software, Inc., Sand Diego, CA, USA). Data representing independently repeated experiments (n=3) were combined and used for further analysis. Collected data passed through Shapiro-Wilk’s normality test, followed by assessing of descriptive statistical characteristic (mean, standard error and standard deviation). One-way analysis of variance (ANOVA), followed by Dunnett’s multiple comparison tests, was used to examine differences between the experimental and control groups. The results were expressed as the mean ± standard error meaning (SEM). P-values equal to or lower than 0.05 were considered statistically significant.

The obtained results in Fig. 1. indicate a slight modification in mitochondrial activity of HUVEC cells, with significant *(p<0.05)* inhibition at 250 μg/mL after 24 h of exposure. The values were established at 92.3±1.17 %. The significant *(p<0.01)* stimulation was recorded at 150 μg/mL (109.00±2.32 %) compared to the control group (100.00±2.30 %). In case of cell membrane integrity authors did not confirm any significant changes compared to the control group (100.00±1.29 %). Individual values fluctuated between 97.02±2.36 % to 105.40±1.52 %. A similar trend was observed for HUVEC lysosomal activity, where none of the concentrations caused significant changes; however, higher doses (250 and 300 μg/mL) indicated a slight decrease varying between 93.51 ± 1.29 % and 92.81 ± 2.05 %, respectively. The release of IL-6 was slightly affected by *Trigonella* microgreens, when higher applied doses (from 150 μg/ml) slightly reduce the release of this cytokine compared to the control group (100.00±1.42 %) (Fig. 2). Taken together, the most significant changes have been recorded in the mitochondrial activity of HUVEC cells induced by higher experimental doses starting from 250 μg/mL. Significant inhibition of this parameter was also confirmed by Iranmenesh [11]. The authors determined *in vitro* effect of *Trigonella foenum-graecum L*. (500–3500 μg/mL) on mitochondrial activity of HUVEC cells, and results showed a significant decrease in mitochondrial activity after 24 h exposure. At the same time, the author states that doses below 500 μg/mL might not have the same progressive effect, which was confirmed by the results from the analyses of the length of vascular tubules formed by HUVEC cells in this study. Inhibition of mitochondrial activity in HUVEC cells treated with *Trigonella* was also observed by [12]. The authors confirmed that experimental doses starting from 200 μg/mL caused a significant decrease in this parameter. It seems that our results are consistent with the claims of previous studies, which suggest the onset of mitochondrial activity inhibition in exposed cells is attributed to the excessively high concentrations applied during the experiment. The same effects were confirmed by studies using pathological cell models such as Hep-2 and MCF-7 tumor cells [13,14]. The authors declare that proliferation could be suppressed by diosgenin through disruption of Ca^+2^ homeostasis and subsequent activation of p53 and modulation of caspase-3 activity. However, these studies also highlight lower sensitivity of healthy cell lines to *Trigonella* treatment compared to carcinoma cell lines *in vitro*.

In addition to the above-mentioned parameters, the release of IL-6 by HUVEC cells was monitored after 24 h exposure to *Trigonella* microgreens. The obtained result revealed a slight decline from 150 to 300 μg/mL (93.01±1.27 %; 94.52±2.37 %) of *Trigonella* microgreen extract, without any significant changes. Thesimilar conclusions were brought by [15]. The gained results confirmed a reduction of cytokines secretions, concretely TNF-α, and IL-6 in THP – 1 macrophage (dTHP-1) stimulated with glycated-bovine serum albumin (BSA). The signalling protein VEGF (vascular endothelial growth factor) plays a significant role as it increases vascular permeability and significantly contributes to inflammation. [12] demonstrated that extracts from *Trigonella foenum-graecum L*. can significantly reduce the expression of VEGF and bFGF (basic fibroblast growth factor) genes, which was confirmed using quantitative RT-PCR method. [16] evaluate *in vitro* effect of *Trigonella-foenum graecum L*. extract on endothelial cells of human coronary artery (HCAEC). Authors clearly demonstrated reduction of gene and protein expression of IL-6 and IL-8 caused by saponins present in *Trigonella*. Moderate differences in cellular responses to Trigonella in the context of cytokine secretion, including IL-6, may be due to the different physiological sensitivity of the individual models, which were confronted with our results mentioned above. In addition, some experimental *in vitro* studies described anti-inflammatory activity of the species such as *Glycine max*, *Medicago sativa*, *Vigna radiata* and *Trigonella* as well, from the *Fabaceae* family. Our results confirmed a slight decrease in IL-6 release without significant changes, while these studies highlight inhibition of IL-1β, TNF-α or IL-6. This may be due to different cell models, prolonged exposure time or different procedures in the preparation of Trigonella extract [17,18]. In conclusion, the results of our pilot study suggest that *Trigonella* microgreen extract can significantly affect mitochondrial activity of HUVEC cells without other alterations in cell membrane integrity and lysosomal activity. Furthermore, we found a slight inhibition of IL-6 release after 24 h of exposure, which, in the context of the detected changes in mitochondrial activity, can cause further decrease of this cytokine with prolonged time of cultivation or with higher Trigonella concentration. In the future, HUVEC cells could become a validated tool in biomedical research in terms of determining the mechanism of action and intracellular interactions related to the inflammatory process and the development of civilization diseases.

**Fig. 1:**
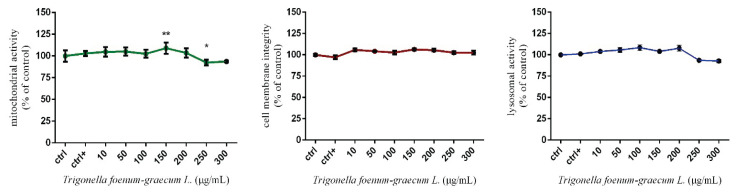
Cellular parameter of HUVEC cells treated by *Trigonella*. ctrl – control group (not treated), ctrl+ – positive control group (zymosan). Each bar represents the mean (±SEM) optical density percent of the control group (ctrl) and microgreen’s extract treated groups. The level of statistical significance was established at **(p<0.01), and *(p<0.05). Statistical differences are indicated by an asterisk

**Fig. 2:**
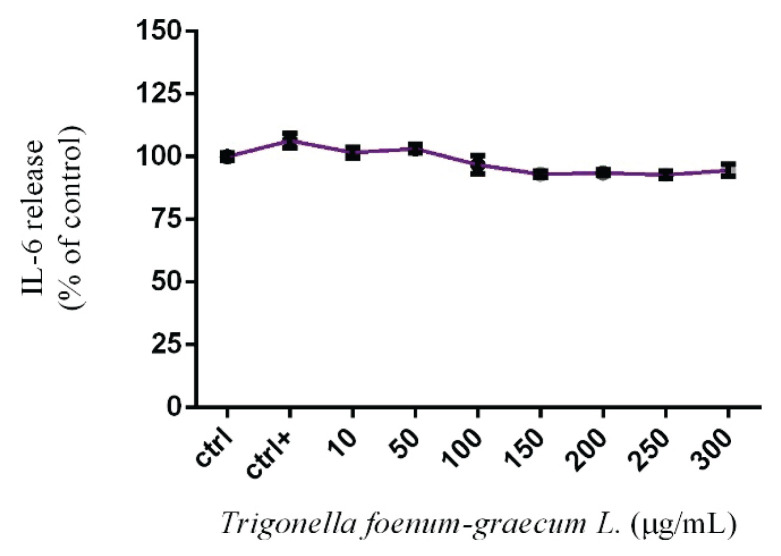
Interleukin – 6 releases by HUVEC cells. ctrl – control group (not treated), ctrl+ – positive control group (zymosan). Each bar represents the mean (±SEM) optical density percent of the control group (ctrl) and microgreen’s extract treated groups.
